# Integration of metabolomics and existing omics data reveals new insights into phytoplasma-induced metabolic reprogramming in host plants

**DOI:** 10.1371/journal.pone.0246203

**Published:** 2021-02-04

**Authors:** Yue Tan, Qingliang Li, Yan Zhao, Hairong Wei, Jiawei Wang, Con Jacyn Baker, Qingzhong Liu, Wei Wei

**Affiliations:** 1 Shandong Institute of Pomology, Taian, China; 2 College of Life Sciences, Zaozhuang University, Zaozhuang, China; 3 United States Department of Agriculture, Molecular Plant Pathology Laboratory, Beltsville Agricultural Research Center, Agricultural Research Service, Beltsville, MD, United States of America; Academia Sinica, TAIWAN

## Abstract

Phytoplasmas are cell wall-less bacteria that induce abnormal plant growth and various diseases, causing severe economic loss. Phytoplasmas are highly dependent on nutrients imported from host cells because they have lost many genes involved in essential metabolic pathways during reductive evolution. However, metabolic crosstalk between phytoplasmas and host plants and the mechanisms of phytoplasma nutrient acquisition remain poorly understood. In this study, using metabolomics approach, sweet cherry virescence (SCV) phytoplasma-induced metabolite alterations in sweet cherry trees were investigated. A total of 676 metabolites were identified in SCV phytoplasma-infected and mock inoculated leaves, of which 187 metabolites were differentially expressed, with an overwhelming majority belonging to carbohydrates, fatty acids/lipids, amino acids, and flavonoids. Available omics data of interactions between plant and phytoplasma were also deciphered and integrated into the present study. The results demonstrated that phytoplasma infection promoted glycolysis and pentose phosphate pathway activities, which provide energy and nutrients, and facilitate biosynthesis of necessary low-molecular metabolites. Our findings indicated that phytoplasma can induce reprograming of plant metabolism to obtain nutrients for its own replication and infection. The findings from this study provide new insight into interactions of host plants and phytoplasmas from a nutrient acquisition perspective.

## Introduction

Phytoplasmas (Acholeplasmatales/*incertae sedis*) are small plant pathogenic bacteria that disrupt growth and development and are associated with numerous diseases in plants including various crops with important agricultural and economic significance [1–5]. In nature, phytoplasmas are transmitted from plants to plants by insect vectors [6]. Residing in plant phloem tissues and being adapted to the nutrient-rich environment, phytoplasmas have gone through reductive evolution, as manifested by lack of many genes that are involved in metabolic pathways essential to free-living organisms, including tricarboxylic acid cycle, pentose phosphate pathway, sterol biosynthesis, fatty acid biosynthesis, *de novo* nucleotide synthesis, and biosynthesis of most amino acids [7]. On the other hand, phytoplasma genomes harbor multiple copies of transporter-related genes such as ABC transporters that can import peptides, amino acids, and nutrients into the cell [7–9]. In onion yellows phytoplasma genome, a total of 27 genes that encode transporters were identified [7]. This strongly indicates that phytoplasmas are highly dependent on metabolites imported from the hosts for their own growth and infection.

Host metabolite changes in response to phytoplasma infection have been reported previously. By using traditional measurement procedures, the accumulations of various carbohydrates, amino acids and total proteins in phytoplasma-infected leaves were unveiled [3,10,11]. For example, in coconut palms infected with lethal yellows phytoplasmas, with the onset of symptoms, the content of sugar and starch in newly expanded and intermediate leaves increased, while the content of sugar and starch in the primary roots decreased significantly in the later stage of infection, indicating that sugar transport through phloem was impaired by phytoplasma infection. Besides traditional biochemical studies, various high-throughput “omics” approaches including transcriptomics, proteomics, and metabolomics were also applied in the studies of host and phytoplasma interactions, revealing the alterations of multiple metabolic pathways mainly sugar metabolism [12,13] and flavonoid biosynthesis [14,15] in host plants upon phytoplasma infection. For instance, a transcriptomic study unveiled that paulownia witches’-broom phytoplasmas affected carbohydrate metabolism of paulownia plants by altering the expression of some host genes involved in cell wall biosynthesis and degradation to meet their energy requirements for growth and spread [13]. In addition, a combined transcriptomics and proteomics study revealed that genes and proteins related to flavonoid biosynthesis and phenylalanine metabolism pathways were up-regulated and accumulated in jujube leaves infected with jujube witches’-broom phytoplasmas [14].

Sweet cherry (*Prunus avium*) is an important fruit tree for both commercial and home growing. In recent years, many sweet cherry trees in China have been observed to exhibit floral virescence, witches’-broom growth, and decline symptoms, some leading to plant death [16–19]. Sweet cherry virescence (SCV) phytoplasmas [group 16SrV, subgroup B (16SrV-B)] have been identified as the etiologic agent for this destructive disease [17–19]. Physiological and transcriptomic analyses have demonstrated that SCV phytoplasma-infected leaves experienced a complex array of metabolic alterations accompanying a source-to-sink functional change, which could profoundly impact the nutrition of both the phytoplasmas and host trees [17,20]. In this study, we took a metabolomics approach to examine SCV phytoplasma induced cellular metabolite changes in leaves of sweet cherry trees. Previous omics data regarding phytoplasma infection were also analyzed, compared, and integrated into the present metabolomics study. The results revealed that phytoplasma infection reprogramed plant metabolisms, induced an elevation in the levels of most differential metabolites that belong to carbohydrates, fatty acids/lipids, and amino acids, and benefit their own growth and infection. The new findings contribute to a better understanding of phytoplasma-plant interactions from a nutritional perspective.

## Materials and methods

### Plant materials and sampling

Sweet cherry trees (*Prunus avium* L. cv. Summit) used for this study were planted in an experimental orchard (36°11’59.6"N/117°11’31.2"E) in suburban Taian, Shandong Province, China. SCV phytoplasma infection was established by graft inoculation using SCV phytoplasma infected sweet cherry branches as scions. Mock inoculation using healthy scions was also carried out. According to the procedures described in our previous study, symptom development was observed, and SCV phytoplasma infection was confirmed by PCR amplification and subsequent sequence analysis [20]. Leaf samples from three SCV phytoplasma-infected seven-year-old sweet cherry trees exhibiting typical witches’-broom symptom and three mock-inoculated healthy controls were used as three independent biological replicates, respectively. In order to monitor the consistency of the entire analytical process, a small aliquot of each biological sample in the study set were pooled together and divided into three quality control (QC) samples. Excised leaf tissues were immediately frozen in liquid nitrogen and kept at -80°C until use.

### Metabolomics experiment procedures

The sample preparation, metabolite extraction, and metabolomics analysis were performed by Wuhan MetWare Biotechnology Co., Ltd. (Wuhan, China), according to their standard procedures and previously described [21–24].

### Sample preparation and metabolite extraction

The frozen leaves were first freeze-dried and then ground to fine powder using a mixer mill (MM 400, Retsch, Germany) with a zirconia bead for 1.5 min at 30 Hz. The powder (100 mg) was treated with 1 ml of 70% aqueous methanol overnight at 4°C. Following centrifugation at 10,000 ×g for 10 min, the extracts in supernatant fraction were absorbed using a CNWBOND Carbon-GCB SPE Cartridge (ANPEL, China) and filtered through a 0.22-μm filter (Millipore, USA) for liquid chromatography-mass spectrometry (LC-MS) analysis [21].

### LC-MS analysis

The LC-MS analysis was conducted as previously described with a few modifications [21,22]. The metabolite extracts were processed by liquid chromatography-electrospray ionization-tandem mass spectrometry (LC-ESI-MS/MS) coupled with a linearity ion-trap (LIT) and triple quadrupole-linear ion trap mass spectrometer (Q TRAP), namely, Q TRAP LC-ESI-MS/MS system (HPLC, Shim-pack UFLC SHIMADZU CBM30A system, Shimadzu, Japan; MS, Applied Biosystems 6500 Q TRAP, Applied Biosystems, USA). Q TRAP LC-ESI-MS/MS system was operated in the positive ion mode and controlled by Analyst 1.6 software (AB Sciex, USA). The analytical conditions and operation parameters of the system were as described previously [21–23].

### Qualitative and quantitative analysis of metabolites

The mass spectrometric (MS) data were collected and processed using Analyst 1.6.1 software (AB Sciex, USA). Qualitative analysis of primary and secondary MS data was performed through comparison with existing mass spectrometry databases (Wuhan Metware Biotechnology Co., Ltd. database, MassBank, KNAPSAcK, HMDB, MoTo DB, and METLIN). The screened data were then processed with MultiQuant software (AB Sciex, USA), and the metabolites were quantitatively analyzed by employing the multiple reaction monitoring mode using triple quadrupole mass spectrometry according to the procures previously described [24].

### Statistical analysis

Hierarchical clustering analysis (HCA), principal component analysis (PCA) and orthogonal partial least squares discriminant analysis (OPLS-DA) are widely used statistical tools for analysis of metabolomics data [25,26]. HCA, also known as hierarchical clustering, is an exploratory tool for clustering analysis in big data research, aiming to reveal the grouping of objects with similar characteristics. PCA, similar to clustering such as HCA, is used to determine whether samples come from different treatment groups. Both HCA and PCA are unsupervised, and suitable for exploratory data analyses, which generate hypotheses rather than verify them. For discriminant (variable) analysis and accurate predictions, OPLS-DA, a supervised model was used. HCA, PCA, and OPLS-DA were conducted as previously described [25,26]. Variable importance in project (VIP) values for each metabolite were generated from the OPLS-DA analysis. |log_2_(fold change)| ≥ 1 and VIP ≥ 1 were set as the thresholds for determination of significantly differential metabolites [22]. Pairwise Pearson correlation coefficients were calculated by R (www.r-project.org/). The Kyoto Encyclopedia of Genes and Genomes (KEGG) database was further used to link differential metabolites to metabolic pathways. *P*-value < 0.05 was selected to reduce the false discovery rate [24].

## Results and discussion

### Identification and categorization of metabolites

Metabolite extracts from both SCV phytoplasma-infected leaves (SCV-IL) and mock inoculated healthy leaves (M-HL) of sweet cherry trees were processed on LC-MS analytical platform. Metabolomics data were collected, processed, and compared with public mass spectrometry databases (See Methods). A total of 676 metabolites were identified in SCV-IL and M-HL samples (S1 Table). These metabolites fall into eight main categories including carbohydrates, fatty acids/lipids, amino acids and their derivatives, nucleotides and their derivatives, phenylpropanoids, flavonoids, organic acids, and other metabolites (Table 1). A high degree of consistency among repeated samples from distinct SCV-IL, M-HL, and quality control (QC) sample groups was confirmed by pairwise Pearson’s correlation coefficient (S1 Fig).

**Table 1:** A summary of identified and differentially regulated metabolites in sweet cherry leaves in response to phytoplasma infection.

Eight main class	Number of metabolites			
	Identified	Differential	Up-regulated	Down-regulated
**Carbohydrates and their derivatives**	**25**	**5**	**5**	**0**
Sugars	7	3	3	0
Phosphorylated sugars	5	2	2	0
Others	13	0	0	0
**Fatty acids/Lipids**	**67**	**20**	**18**	**2**
Fatty acids	17	6	5	1
Sphingolipid	1	1	1	0
Glycerolipids	17	9	8	1
Glycerophospholipids	32	4	4	0
**Amino acids and their derivatives**	**82**	**14**	**7**	**7**
Proteinogenic amino acids	19	4	3	1
Non-proteinogenic amino acids	10	4	1	3
Amino acid derivatives	53	6	3	3
**Nucleotides and their derivatives**	**55**	**11**	**4**	**7**
Components of nucleic acids	19	4	0	4
Others	36	6	3	3
**Phenylpropanoids**	**84**	**35**	**19**	**16**
Cinnamic acid and its derivatives	50	19	11	8
Coumarin and its derivatives	34	16	8	8
**Flavonoids**	**183**	**65**	**46**	**19**
Flavone	56	15	11	4
Flavonol	38	15	9	6
Flavone C-glycosides	25	9	7	2
Flavanone	21	6	3	3
Anthocyanins	14	6	5	1
Isoflavone	12	3	2	1
Catechin and its derivatives	10	5	5	0
Proanthocyanidins	5	5	4	1
Flavonolignan	2	1	0	1
**Organic acids**	**85**	**19**	**10**	**9**
**Other metabolites**	**95**	**19**	**10**	**9**
**Total**	**676**	**187**	**118**	**69**

### Clustering of differentially accumulated metabolites

**Fig 1:**
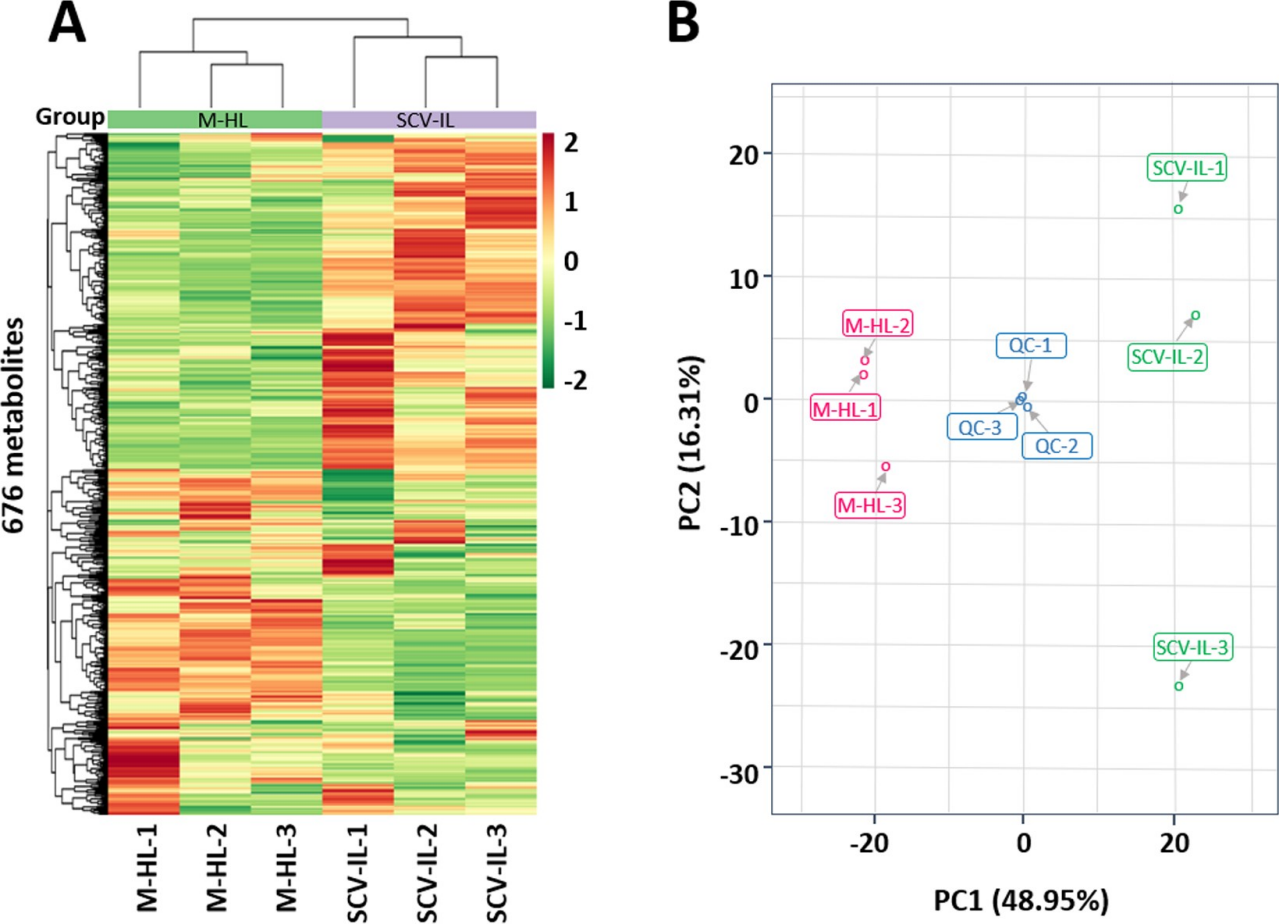
Hierarchical clustering analysis (HCA) and principal component analysis (PCA) of the metabolic data derived from the metabolites of mock healthy leaf (M-HL) and SCV phytoplasma-infected leaf (SCV-IL) samples of sweet cherry trees. (A) Heatmap visualization of the HCA based on the relative level of identified 676 metabolites among M-HL and SCV-IL leaf samples. The rows show differential metabolites, and the columns represent samples M-HL and SCV-IL; the color spectrum depicts relative abundance. Red and green indicate high and low abundance, respectively. The color scale key is shown on the right of the heatmap. (B) PCA score plot for M-HL, SCV-IL, and quality control (QC) samples.

Metabolomic studies combined with statistical analysis have been widely employed for characterization of similarly or differentially accumulated metabolites between/among different sample groups [27]. In this study, unsupervised statistical analyses including hierarchical cluster analysis (HCA), and principal component analysis (PCA) were initially performed to obtain an overview of the metabolic similarity or difference among SCV-IL and M-HL sample groups. HCA results revealed that SCV-IL and M-HL were clearly divided into two distinct clusters (Fig 1A). Consistent with HCA results, SCV-IL and M-HL were also distinguished into two groups by PCA plot analysis. The interpreted values of PC1 and PC2 were 48.95% and 16.31%, respectively. In addition, the PCA score of each QC sample was close to that of two other QC samples, namely, the QC samples were tightly clustered (Fig 1B), demonstrating the technical stability and consistency of the entire analytical process. The results from both HCA and PCA manifested that SCV-IL and M-HL samples have mutually distinct metabolic profiles.

**Fig 2:**
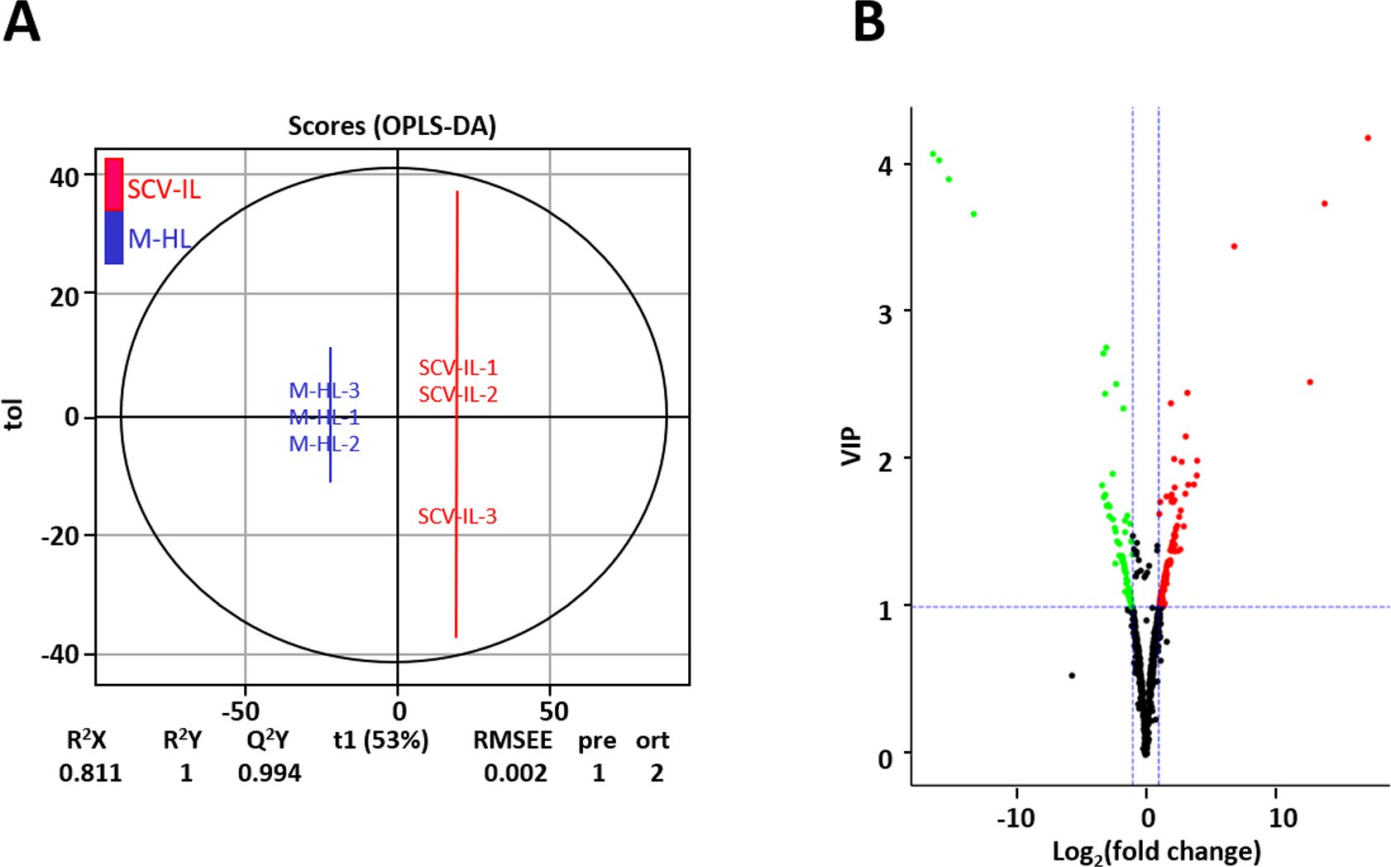
OPLS-DA score plots (panel A) and the corresponding volcano plots (panel B) derived from differential metabolites of mock healthy leaf (M-HL) and SCV phytoplasma-infected leaf (SCV-IL) samples of sweet cherry trees. In panel B, red, green and black dots respectively represent up-regulated, down-regulated and unchanged metabolites between M-HL and SCV-IL samples.

Subsequently, a supervised statistical analysis, orthogonal partial least squares-discriminant analysis (OPLS-DA), was employed to quantitatively differentiate metabolites levels between SCV-IL and M-HL samples. As shown in Fig 2A, the goodness of fit parameters (R^2^X = 0.811, R^2^Y = 1) and the predictive ability parameter (Q^2^ = 0.994) clearly separated SCV-IL and M-HL samples into two different clusters, another strong indicator of significant differences in metabolite levels between SCV-IL and M-HL samples.

To gain deeper insight into the metabolic differences between SCV-IL and M-HL samples, differential metabolites were screened based on the fold-change and the VIP values that were generated by the OPLS-DA model. Using the cutoff criteria VIP ≥ 1 and |log_2_ (fold change)| ≥ 1, 187 differential metabolites were identified from initially detected 676 common metabolites in SCV-IL and M-HL samples (S2 Table). Among them, 118 metabolites were upregulated, while 69 were downregulated in SCV-IL vs. M-HL samples (S2 Table). The statistical significance versus the magnitude of change of the upregulated, downregulated, and unchanged metabolites are graphically illustrated in using a volcano plot (Fig 2B).

### Classification and enrichment of the differential metabolites

187 differentially accumulated metabolites between SCV-IL and M-HL samples were further mapped to the KEGG database (http://www.genome.jp/kegg/) to obtain detailed pathway information. Most of the differential metabolites were upregulated in leaves of sweet cherry trees upon phytoplasma infection, involving in carbohydrate and fatty acid/lipid biosynthesis pathways and flavonoid signaling pathways. Details of the differential metabolites are shown in S2 Table.

### Integration of previous omics data into the current study

Various high-throughput “omics” approaches developed in the recent years such as transcriptomics (quantitative analysis of mRNA transcripts), proteomics (analysis of protein composition), and metabolomics (identification and quantification of cellular metabolites) have been widely employed in the study of plant-phytoplasma interactions. So far, a total 23 of omics studies targeting phytoplasma-plant interactions have been reported, which include 10 transcriptomics [13,20,28–35], 7 proteomics [36–42], 3 metabolomics [43–45], and 3 mixed omics studies [14,15,46]. These studies mainly focused on Bois Noir (BN), Flavescence dorée (FD), paulownia witches’-broom (PaWB), and jujube witches’-broom (JWB) phytoplasma-infected grapevine, paulownia, and jujube plants. To gain a more comprehensive understanding of plant-phytoplasma interactions, the new metabolomics data from SCV phytoplasma-infected sweet cherry tree (present study) were compared with existing omics data (previous reports), and the results were summarized as shown in S3 Table.

### Changes in carbohydrates

**Fig 3:**
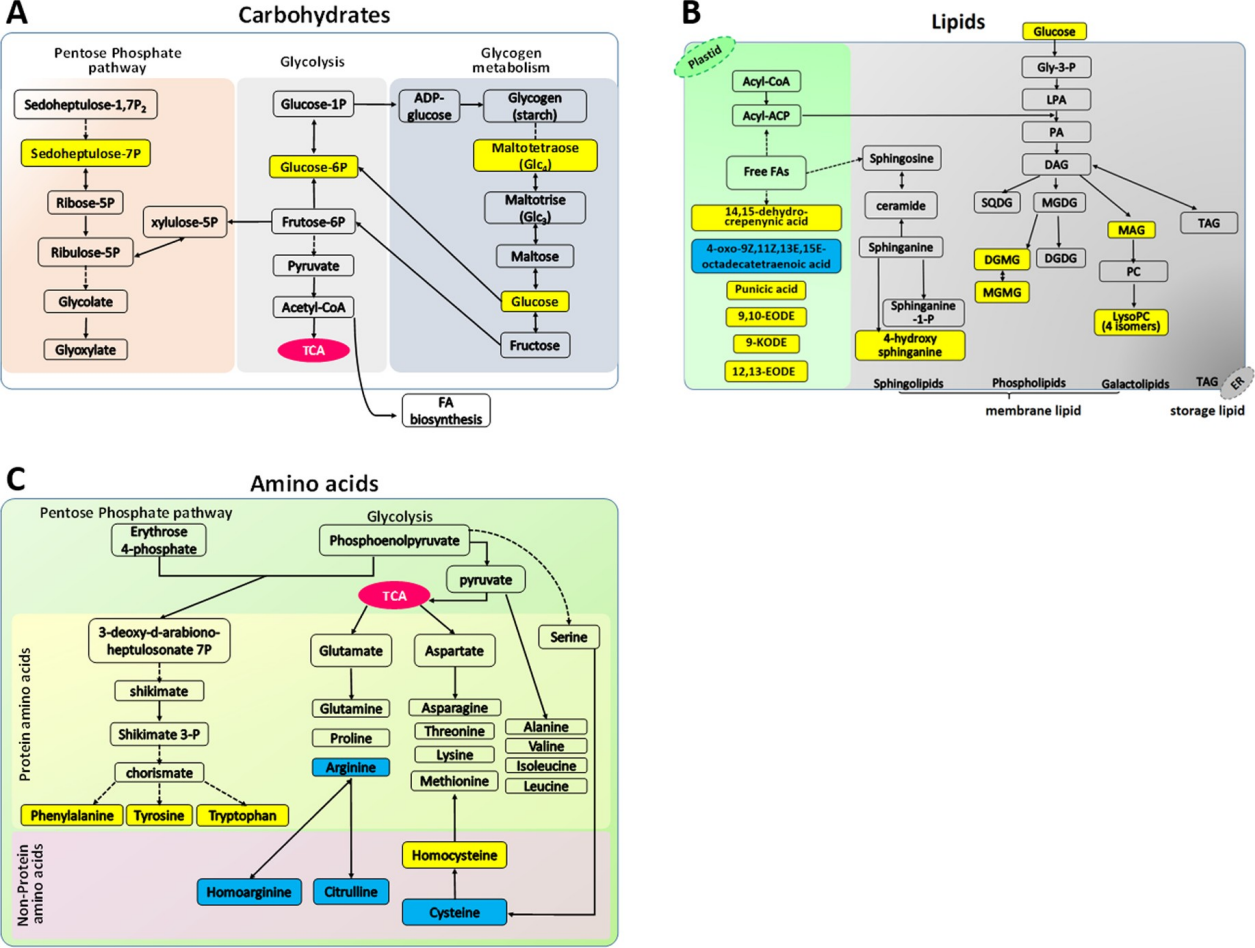
Sweet cherry virescence (SCV) phytoplasma infection induced major metabolite alterations (A-C) in sweet cherry trees. (A): Carbohydrate metabolites, (B): FA/lipid metabolites, and (C) amino acid metabolites. Yellow and blue boxes represent up-regulated and down-regulated metabolites in SCV phytoplasma infected leaf samples comparing with mock inoculated healthy leaf samples. ACP: ACyl carrier protein; CoA: Coenzyme A; DAG: Diacylglycerol; DGDG: Digalactosyl diglyceride; DGMG: Diglycosyl monoacylglycerol; EODE: Epoxyoctadecadienoic acid; ER: Endoplasmic reticulum; FA: Fatty acid; Gly-3-P: Glycerol-3-phosphate; KODE: Oxooctadecadienoic acid; LPA: Lysophosphatidic acid; LysoPC: Lysophosphatidylcholine; MAG: Monoacylglycerol; MGDG: Monogalactosyl diglyceride; MGMG: Monoglycosyl monoacylglycerol; PA: Phosphatidic acid; PC: Phosphatidylcholine; SQDG: Sulphoquinovosyl diglyceride; TAG: Triacylglycerol; TCA: Tricarboxylic acid cycle.

A total of twelve sugars and phosphorylated sugars were identified in SCV-IL and M-IL samples (Table 1). Among them, five were up-regulated in SCV-IL samples comparing with M-HL samples, while none of them were down-regulated (Tables 1 and 2). The upregulated metabolites included D-glucose, D-glucose 6-phosphate, D-sedoheptulose 7-phosphate, maltotetraose and D-melezitose (Table 2 and Fig 3A).

**Table 2:** Identification of differentially regulated metabolites in sweet cherry leaves in response to phytoplasma infection by orthogonal partial least squares discriminant analysis (OPLS-DA) based on fold changes and VIP values.

Class	Subclass	Compound	Compound Index	Fold change	VIP value
Carbohydrates		D-Glucose	pme1846	3.16	1.28
		D-Glucose 6-phosphate	pme3160	5	1.53
		D-Sedoheptuiose 7-phosphate	pme3163	2.2	1.05
		D-Melezitose	pme0500	3.49	1.31
		Maltotetraose	pmb2858	2.97	1.24
Fatty acids/lipids	Fatty acids	14,15-Dehydrocrepenynic acid	pma0461	2.47	1.14
		4-Oxo-9Z,11Z,13E,15E-octadecatetraenoic acid	pmb0885	0.44	1.07
		Punicic acid	pmb0889	2.12	1.04
		9,10-EODE	pmb2778	2.06	1
		9-KODE	pmb2787	3.12	1.29
		12,13-EODE	pmb2799	2.74	1.19
	Sphingolipid	4-Hydroxysphinganine	pmb2221	3.94	1.71
	Simple glycerolipids	MAG (18:1) isomer1	pmb2363	3.98	1.72
		MAG (18:2)	pmb0890	2.18	1.06
		MAG (18:3) isomer5	pmb0160	2.6	1.13
		MAG (18:4) isomer3	pmb1562	0.33	1.58
	Glyceroglycolipids	MGMG (18:2) isomer2	pmb2383	2.84	1.21
		DGMG (18:1)	pmb2251	2.51	1.1
		DGMG (18:2) isomer1	pmb0161	2.91	1.23
		DGMG (18:2) isomer2	pmb0159	2.41	1.08
		DGMG (18:2) isomer3	pmb0163	2.95	1.24
	Glycerophospholipids	LysoPC 16:1 (2n isomer)	pmb0848	2.56	1.17
		LysoPC 16:2 (2n isomer)	pmb0863	2.35	1.07
		LysoPC 18:3 (2n isomer)	pmb0865	2.12	1.04
		LysoPC 18:2 (2n isomer)	pmb0873	2.72	1.21
Amino acids	Proteinogenic amino acids	L-Phenylalanine	pme0021	2.76	1.19
		L-Arginine	pme0042	0.11	1.74
		L-Tryptophan	pme0050	4.86	1.48
		L-Tyrosine	pme1070	3.7	1.38
	Non-proteinogenic amino acids	L-Citrulline	pme0007	0.28	1.31
		L-Cystine	pme0016	0.41	1.12
		DL-Homocysteine	pme0057	4.09	1.39
		Homoarginine	pme3388	0.23	1.43
Nucleotides and their derivatives	Nucleosides	Adenosine	pmd0023	0.29	1.34
		Uridine	pme1063	0.44	1.06
		Cytidine	pme3732	0.3	1.33
	Nucleotides	2’-Deoxycytidine-5’-monophosphate	pme1373	0.3	2.34
		3’,5’-Cyclic adenosine monophosphate	pmb2684	0.32	1.29
		3’,5’-Cyclic guanosine monophosphate	pme3835	3.02	1.27
	Others	6-Methylthiopurine	pmc0274	2.68	1.15
		Adenosine O-ribose	pmc0281	0.4	1.13
		Hypoxanthine	pme0033	0.47	1.05
		Xanthine	pme0256	2.39	1.11

EODE, epoxyoctadecadienoic acid; KODE, oxooctadecadienoic acid; MAG, monoacylglycerol; MGMG, monoglycosyl monoacylglycerol; DGMG, diglycosyl monoacylglycerol; LysoPC, lysophosphatidylcholine.

D-glucose 6-phosphate is the entry molecule of glycolysis, that is the first step in the breakdown of glucose to produce energy in the form of ATP. Both phytoplasma proliferation and disease induction in host plants require high amounts of energy. Maltotetraose, a linear tetramer of α-D-glucose, is one of the intermediate products during the starch breakdown, and is cleaved mainly to two maltose molecules, but also to one maltotriose and one glucose [47]. Elevated levels of maltotetraose, glucose, and glucose 6-phosphate in SCV-IL samples may indicate an enhancement of glycolysis in sweet cherry trees upon phytoplasma infection. This result is consistent with the upregulated expression of glycolytic genes in our previous transcriptomic analysis [20] and other existing omics data (S3 Table).

It remains unclear how phytoplasma import sugar and generate glucose-6-phosphate to support the glycolysis pathway since phytoplasma genomes lack phosphotransferase system and sucrose degrading enzymes that play important roles in sugar transport and utilization. However, phytoplasmas possess a variety of ABC transporters. Functional modulation of ABC transporter activities is considered one of the primary strategies for pathogens to obtain nutrients from living host cells or other nutrient niches. For example, ABC transporters in plant-pathogenic *Pseudomonas* (Pseudomonadales/*Pseudomonadaceae*) species facilitate the uptake of maltose, glucose, and sucrose [48]. In addition, the maltose/trehalose ABC transporter in some bacteria recognizes not only maltose and trehalose, but also sucrose and palatinose [49], therefore, it has been proposed that phytoplasma may utilize sucrose and trehalose (the main sugars in plant phloem and insect hemolymph) to enter glycolysis [50]. Maltotetraose is hydrolyzed to maltose by α-amylase [47]. Genes that encode α-amylase were mostly up-regulated in plants upon phytoplasma infection, such as BN and PaWB phytoplasma-infected grapevine and paulownia [29] (existing transcriptomics data, see S3 Table). The up-regulation of maltotetraose in SCV phytoplasma-infected sweet cherry trees indicates that maltose may also act a carbon supply for phytoplasma growth.

Sedoheptulose 7-phosphate is an intermediate metabolite of photosynthesis and the pentose phosphate pathway (PPP). Sedoheptulose-1,7-bisphosphatase (SPBase), an enzyme that catalyzes the removal of a phosphate group from sedoheptulose 1,7-bisphosphate to produce sedoheptulose 7-phosphate [51]. Based on existing omics data, SPBase activity decreased in mulberry dwarf phytoplasma-infected mulberry leaves [37] (S3 Table). As a product of SPBase, enhanced sedoheptulose 7-phosphate may inhibit SPBase activity by negative feedback, a key regulatory feature of metabolism. Besides elevated sedoheptulose 7-phosphate, expression of the gene encoding glucose-6-phosphate 1-dehydrogenase (a key enzyme of the PPP) was also upregulated in SCV phytoplasma-infected leaves of sweet cherry trees based on our previous transcriptomics study [20]. These data pointed to a phytoplasma-induced increase in PPP activity in host plants. PPP is crucial for the biosynthesis of multiple primary low molecular metabolites. This pathway provides nicotinamide adenine dinucleotide phosphate (NADPH) for the biosynthesis of fatty acids, erythrose 4-phosphate for the biosynthesis aromatic amino acids, and pentose for the synthesis of nucleotides. Since phytoplasmas do not possess a PPP, nor the ability to synthesize many essential compounds required for free-living cells [7,52], the enhanced PPP activity in infected plants may be a manifestation of phytoplasma nutrient demand.

Melezitose is a component of honeydew that acts as an attractant or food for insects [53]. This compound also benefits insects by reducing osmotic stress [54]. An upregulation of melezitose in SCV phytoplasma-infected sweet cherry trees might increase the attractiveness to plant sap-sucking insects, potential vectors for phytoplasma transmission. Plant viruses are known to manipulate the visual or odor cues of infected plants to change insect vectors’ feeding behavior, thereby enhancing their spread [55]. In addition, phytoplasma infection can also induce enhanced insect vector reproduction [56]. Phytoplasma infection-induced elevation in melezitose levels strongly suggests that phytoplasmas are capable of modulating plant metabolisms to benefit their own survival and transmission. Further study on the role of melezitose in phytoplasma infected plants and insect vectors will help us to understand the mechanism of phytoplasma transmission and possible disease management strategies.

### Changes in fatty acids/lipids

Fatty acids are basic building blocks for assembly of membrane lipids that are essential throughout life cycle of any cellular organism. In plants, fatty acids are synthesized in plastids and transported to the endoplasmic reticulum (ER) for modification and lipid assembly [57]. Phytoplasmas do not possess a pathway for *de novo* fatty acid synthesis, therefore, the crucial components for membrane lipid assembly of phytoplasma cells are conceivably to be imported from host plants [7].

In this study, a total of 67 lipid-related metabolites were identified in both SCV-IL and M-HL samples, 20 of which (29.85%) were differentially accumulated. Such differential lipids include six fatty acids, one sphingolipid, four simple glycerolipids, five glyceroglycolipids and four glycerophospholipids (Table 2). They were mostly upregulated, accounting for 90% (Table 2 and Fig 3B).

The differential fatty acids identified in the present study were all polyunsaturated (Table 2), and their roles in phytoplasma-infected plants kept unknown. Accumulation of 9-oxooctadecadienoic acid (9-KODE) was observed in rice leaves in response to jasmonic acid (JA) treatment and pathogen infection [58]. Exogenous application of 9-KODEs to rice leaves also induced accumulation of the defensive secondary metabolites such as sakuranetin, naringenin, and serotonin [58]. 9-KODE was also found during soybean germination under microbial stress [59]. Therefore, upregulation of 9-KODE in SCV-IL samples may be related to natural defense responses in sweet cherry trees against phytoplasma infection.

All 14 differentially regulated lipid metabolites identified in the present study were membrane-related, including one sphingolipid (4-Hydroxysphinganine), four simple glycerolipids [monoacylglycerols (MAGs)], five glyceroglycolipids [one monoglycosyl monoacylglycerol (MGMG) and four diglycosyl monoacylglycerols (DGMGs)], and four glycerophospholipids [lysophosphatidylcholines (LysoPCs)]. 4-Hydroxysphinganine is an intermediate in sphingolipid metabolism pathway (Fig 3B) and is present in many plant tissues. MGMG and DGMG are minor glycolipids in plants, relative to major lipids monogalactosyl diglyceride (MGDG) and digalactosyl diglyceride (DGDG) [60]. So far, very few studies on MGMG and DGMG have been reported. Monoacylglycerols (MAGs) are produced not only in plants, but also in bacteria. Using monoacylglycerol intermediates, diacylglycerols can be hydrolyzed to glycerol and free fatty acids by bacterial diacylglycerol lipases [61]. The roles of MGMG and DGMG in phytoplasma-infected plants remain unknown.

LysoPCs are present as minor phospholipids in the cell membrane [62]. They are derived from hydrolysis of phosphatidylcholines by phospholipase, an enzyme that plays an important role in changing the composition of membrane lipids [63]. The level of LysoPC increases in plants when they are under abiotic stress [63]. Accumulation of LysoPCs also promotes dissolution of the cell membrane in human cells [64]. Therefore, the increased LysoPC level in SCV-IL samples may induce alterations in the composition, the permeability, and even degradation of host plant cell membrane. It would be interesting to learn whether such host membrane alterations play a role in increasing nutrient transport from host cells to phytoplasmas and facilitating the growth and replication of phytoplasmas.

Our finding of increased LysoPCs in SCV-IL samples was also consistent with existing omics data. In both apple proliferation phytoplasma-infected tobaccos [46] (proteomics/transcriptomics data), and PaWB phytoplasma-infected paulownia plants [29] (transcriptomics data), phospholipase for LysoPC synthesis was upregulated (S3 Table). Besides plant phospholipase, phytoplasmal phospholipase could also participate in host membrane dissolution. Phospholipase has been identified in Malaysian periwinkle yellow phytoplasma, and its lipolytic activity has been functionally verified in both *E*. *coli* and yeast [65].

It is well known that the composition of bacterial membrane lipids is different from that of plant membrane lipids [66,67]. There are different types of lipids in plants including triacylglycerols, phospholipids, galactolipids, and sphingolipids. While triacylglycerols are essentially neutral storage lipids, phospholipids, galactolipids, and sphingolipids are important membrane lipids [67,68]. The elevated lipid metabolites found in the SCV phytoplasma-infected sweet cherry leaves were all membrane-related lipids (Fig 3B and Table 2). In this study, bacteria-specific lipid metabolites were not identified in SCV-IL samples. However, based on existing omics data, ornithine was found in BN phytoplasma-infected grapevine [32] (Transcriptomics data in S3 Table). Ornithine-containing lipids are widely present in bacteria but absent from eukaryotes [66]. Such data indicate that phytoplasma is capable of utilizing host-derived components for its own lipid assembly. Further studies are needed to elucidate the pathway of lipid trafficking and membrane assembly in phytoplasmas.

Our present study revealed that, in SCV-IL samples, 90% of differentially regulated lipid metabolites were increased, indicating that phytoplasma infection enhanced lipid metabolism of host plants. This finding is also strongly supported by existing omics data. Our previous transcriptomic study (S3 Table) showed that, in sweet cherry, multiple genes associated with fatty acid biosynthesis were upregulated in response to SCV phytoplasma infection [20]. Similarly, a series of genes involved in fatty acid biosynthesis, degradation, and metabolism were also upregulated in Mexican lime leaves that were infected with ‘*Candidatus* Phytoplasma aurantifolia’ [33].

### Changes in amino acids

Amino acids play essential roles in plants. They not only provide building blocks for protein synthesis, but also participate in various processes of plant growth, development, and homeostasis [69]. Since many genes that encode enzymes required for amino acid biosynthesis are absent in phytoplasma genomes, amino acids for phytoplasma growth and replication are believed to be imported from host cells [7,52].

Our previous transcriptomic data showed that, in sweet cherry leaves, the SCV phytoplasma infection altered host amino acid metabolism in a complex manner [20]. In the present metabolomic study, nineteen proteinogenic and ten non-proteinogenic free amino acids were identified in both SCV-IL and M-HL samples. However, among them, only four proteinogenic amino acids (phenylalanine, tyrosine, tryptophan, and arginine) and four non-proteinogenic amino acids (homocysteine, homoarginine, citrulline, and cysteine) were differentially accumulated between SCV-IL and M-HL samples (Tables 1 and 2). This result indicated that, in leaves of the SCV phytoplasma-infected sweet cherry trees, the levels of most proteinogenic amino acids (15 out of 19, 78.9%) remained unchanged under the infection conditions. While such result appeared contrary to the hypothesis of phytoplasma’s dependence on host-derived amino acids for protein synthesis, a possible explanation could be as follows: most proteinogenic amino acids were produced by the host “on-demand” and were promptly utilized by phytoplasmas; as a result, the host amino acid homeostasis was maintained.

Notably, three aromatic proteinogenic amino acids (phenylalanine, tyrosine, and tryptophan) were all upregulated in SCV-IL samples, indicating the enhancement of the shikimate pathway (Fig 3C). The shikimate pathway generates aromatic amino acids using phosphoenolpyruvate and erythrose 4-phosphate as primary substrates, that are generated by glycolysis and the PPP, respectively [70]. The enhancement of this pathway was consistent with the enhanced glycolysis and PPP in phytoplasma-infected plants (this study and previous reports [19,20]).

Furthermore, phenylalanine and tyrosine are essential for the biosynthesis of various polyphenol compounds via the phenylpropanoid pathway. Phenylalanine ammonia lyase catalyzes the non-oxidative deamination of L-phenylalanine into cinnamic acid [71], which is the first step in this pathway [72]. The increase in the levels of L-phenylalanine and cinnamic acid (Table 2) in phytoplasma-infected leaves was indicative of an enhancement of phenylpropanoid pathway, leading to the accumulation of various phenylpropanoids and flavonoids.

Tryptophan is an important precursor of indole-3-acetic acid (IAA), the most common member of naturally occurring auxins [73]. No significant change in IAA content was found in mature leaves of the SCV phytoplasma-infected sweet cherry trees according to a previous study [17]. Tryptophan increase in the present study may be a result of the enhanced shikimate pathway.

L-arginine and its metabolism play an important role in plant biology. In addition to being an essential amino acid for protein synthesis, L-arginine also acts as a precursor for the formation of multiple bioactive compounds, such as polyamines, plant alkaloids, and nitric oxide (NO) [73–76]. NO is a major player in plant resistance to pathogens, affecting both basal defense and hypersensitive response [77–79]. Similar to mammals, NO is synthesized from L-arginine, and yields L-citrulline as a byproduct in plants [80–82]. In this study, L-arginine and L-citrulline were both reduced in SCV-IL samples, and L-homoarginine, another derivative of L-arginine [83], was also decreased (Table 2 and Fig 3C), indicating inhibited NO production. It remains to be elucidated whether suppressed NO production will be beneficial to phytoplasma replication in plants.

### Changes in nucleotides and their derivatives

Phytoplasmas possess no genetic repertoire for *de novo* synthesis of nucleotides; instead, these organisms contain genes for the salvage pathway for purine and pyrimidine metabolism [7,8]. Because no nucleobase or nucleoside transporter has been identified in phytoplasmas to date [7,8], these organisms, like many other mollicutes, may depend on nucleotide precursors from host cells [84].

In this study, we found that the levels of three nucleosides and one nucleotide that are components of nucleic acids decreased in phytoplasma-infected leaves (Table 2). However, xanthine, a product of purine degradation, increased (Table 2). This finding suggests enhanced nucleotide degradation, which might provide the substrate via salvage pathway for biosynthesis of phytoplasmas nucleotides. Since the mechanism of nucleotide acquisition by phytoplasma is still unknown, the relationship between host nucleotide metabolism and phytoplasma nutrition needs to be further explored.

Cyclic adenosine monophosphate (cAMP) and cyclic guanosine monophosphate (cGMP) are important second messengers for intracellular signal transduction [85]. The biological activity of these molecules, especially cGMP, has been well documented in a range of plant biological processes [86]. In this study, these two nucleotides were differentially regulated in sweet cherry leaves upon phytoplasma infection (Table 2). cGMP has been implicated to involve in the plant response to pathogen infections [87] as well as signal transduction of hormones such as gibberellins [88], abscisic acid [89] and brassinolides [90]. Considering that phytoplasma infection seriously perturbs the hormone balance and signal transduction of host plants [91], cGMP may be involved in the interactions between phytoplasma and host plants.

### Changes in phenylpropanoids and flavonoids

Flavonoids play very important roles in plant resistance against pathogenic bacteria and fungi [92]. Flavonoids are derived from phenylpropanoids [93]. During the biosynthesis of phenylpropanoid, phenylalanine/tyrosine ammonia lyase converts L-phenylalanine and L-tyrosine into cinnamic acid and *p*-coumaric acid, respectively. Via catalysis by a series of enzymes, *trans*-cinnamic acid can be successively transformed to *p*-coumaric acid, 4-coumaroyl-CoA and finally to chalcone, the precursor of all flavonoids [71].

In this study, we found that 70.8% (46 out of 65) of the differentially regulated flavonoids in SCV-IL samples were upregulated (Table 1). Although *p*-coumaric acid, 4-coumaroyl-CoA and chalcones were not identified in our metabolomic study, levels of cinnamic acid and most *p*-coumaric acid derivatives were elevated in plants upon phytoplasma infection (Table 2). This finding, along with reduced levels of most other phenylpropanoids, may reflect phytoplasma-induced activation of *p*-coumaric acid biosynthesis and metabolism, which contributes to flavonoid biosynthesis.

According to the available omics data, the accumulation of flavonoids and the activation of genes involved in flavonoid biosynthesis have also been found in multiple phytoplasma-infected plants (S3 Table), such as paulownia (*Paulownia tomentosa*) [29,30], grapevine (*Vitis vinifera*) [15,28,39], Mexican lime (*Citrus × aurantiifolia*) [33], jujube (*Ziziphus jujuba*) [14,34] and coconut (*Cocos nucifera*) [35]. Increased flavonoid synthesis in phytoplasma-infected plants may be part of natural plant defensive response against pathogen infections.

## Conclusions

Under phytoplasma infection conditions, the metabolic networks of the invading pathogen and its host are interconnected. Since phytoplasma acquires nutrients from its host, it must compete with the host for similar or identical nutrient substrates within the microenvironment, and a slight alteration in metabolism could significantly affect the outcome of the pathogen-host interactions. Phytoplasmas exclusively inhabit the sieve cells of phloem, and lack many genes involved in the metabolic pathways essential for free-living cells. Thus, phytoplasmas are considered necessary to modulate the metabolism of host plant cells for the supply of nutrients, energy, and metabolites to establish successful replication and infection in plants. By employing metabolomics approach and integrating existing omics data, the present study revealed that phytoplasma infection promoted glycolysis and increased PPP activity in plants. Enhanced glycolysis and PPP activity not only provided energy and nutrient substances, but also facilitated biosynthesis of necessary low molecular metabolites, including amino acids, nucleotides, and fatty acids/lipids, which are conducive to phytoplasma replication and infection. The host plant relies on the similar nutrient substrates and low molecular metabolites within the same microenvironment to support host defense response to phytoplasma infection. A prime example is enhanced flavonoid biosynthesis upon phytoplasma infection. Phytoplasma infection also led to the accumulation of a compound that attracts phloem sap-sucking insects, which is beneficial to the survival and transmission of phytoplasma. These findings indicate that phytoplasma can induce metabolic reprogramming in host plants to favor its own growth and infection.

## Supporting information

S1 Fig A clustered heatmap of pairwise Pearson’s correlation coefficients of the metabolites from three replicates of mock healthy leaf (M-HL) and SCV phytoplasma-infected leaf (SCV-IL), and quality control (QC) samples of sweet cherry trees. Color key scale is shown on the right of the heatmap.
(PPTX)

S1 Table Details of all metabolites identified in SCV-IL M-HL and QC samples. (XLSX)

S2 Table Details of the differential metabolites identified between SCV-IL and M-HL samples. (XLSX)

S3 Table A summary of existing omics data of host plants in response to phytoplasma infection. (Non-highlighted cell and highlighted cell in blue represents up-regulated and down-regulated genes protein, or metabolite, respectively. Highlighted cell in yellow represent gene, protein, or metabolite that both up-and down-regulated).
(XLSX)

## References

[1] Bertaccini A., Duduk B., Paltrinieri S. and Contaldo N. Phytoplasmas and phytoplasma diseases: a severe threat to agriculture. 2014; Am. J. Plant Sci., 2014 10.4236/ajps.2014.53037 26167393 PMC4498585

[2] Jiang H., Wei W., Saiki T., Kawakita H., Watanabe K. and Sato M.. Distribution patterns of mulberry dwarf phytoplasma in reproductive organs, winter buds, and roots of mulberry trees. J. Gen. Plant Pathol. 2004; 70: 168–173.

[3] Maust B.E., Espadas F., Talavera C., Aguilar M., Santamaría J.M. and Oropeza C. Changes in carbohydrate metabolism in coconut palms infected with the lethal yellowing phytoplasma. Phytopathology 2003; 93: 976–981. 10.1094/PHYTO.2003.93.8.976 18943864

[4] Wu Y., Hao X., Li Z., Gu P., An F., Xiang J., et al Identification of the phytoplasma associated with wheat blue dwarf disease in China. Plant Dis. 2010; 94: 977–985. 10.1094/PDIS-94-8-0977 30743487

[5] Wei W., Davis R.E., Nuss D.L. and Zhao Y. Phytoplasmal infection derails genetically preprogrammed meristem fate and alters plant architecture. Proc. Natl. Acad. Sci. U.S.A. 2013; 110: 19149–19154. 10.1073/pnas.1318489110 24191032 PMC3839765

[6] Weintraub P.G. and Beanland L. Insect vectors of phytoplasmas. Annu. Rev. Entomol. 2006; 51: 91–111. 10.1146/annurev.ento.51.110104.151039 16332205

[7] Oshima K., Kakizawa S., Nishigawa H., Jung H.Y., Wei W., Suzuki S., et al Reductive evolution suggested from the complete genome sequence of a plant-pathogenic phytoplasma. Nat. Genet. 2004; 36: 27–29. 10.1038/ng1277 14661021

[8] Bai X., Zhang J., Ewing A., Miller S.A., Radek A.J., Shevchenko D.V., et al Living with genome instability: the adaptation of phytoplasmas to diverse environments of their insect and plant hosts. J. Bacteriol. 2006; 188: 3682–3696. 10.1128/JB.188.10.3682-3696.2006 16672622 PMC1482866

[9] Oshima K., Maejima K. and Namba S. Genomic and evolutionary aspects of phytoplasmas. Front Microbiol. 2013; 4: 230 10.3389/fmicb.2013.00230 23966988 PMC3743221

[10] Junqueira A., Bedendo I. and Pascholati S. Biochemical changes in corn plants infected by the maize bushy stunt phytoplasma. Physiol. Mol. Plant Pathol. 2004; 65: 181–185.

[11] Lepka P., Stitt M., Moll E. and Seemüller E. Effect of phytoplasmal infection on concentration and translocation of carbohydrates and amino acids in periwinkle and tobacco. Physiol. Mol. Plant Pathol. 1999; 55: 59–68.

[12] Monavarfeshani A., Mirzaei M., Sarhadi E., Amirkhani A., Khayam Nekouei M., Haynes P.A., et al Shotgun proteomic analysis of the Mexican lime tree infected with "Candidatus Phytoplasma aurantifolia". J. Proteome Res. 2013; 12: 785–795. 10.1021/pr300865t 23244174

[13] Mou H.Q., Lu J., Zhu S.F., Lin C.L., Tian G.Z., Xu X., et al Transcriptomic analysis of Paulownia infected by Paulownia witches’-broom Phytoplasma. PLoS One 2013; 8: e77217 10.1371/journal.pone.0077217 24130859 PMC3795066

[14] Ye X., Wang H., Chen P., Fu B., Zhang M., Li J., et al Combination of iTRAQ proteomics and RNA-seq transcriptomics reveals multiple levels of regulation in phytoplasma-infected Ziziphus jujuba Mill. Hortic. Res. 2017; 4: 17080 10.1038/hortres.2017.80 29285398 PMC5744194

[15] Margaria P., Ferrandino A., Caciagli P., Kedrina O., Schubert A. and Palmano S. Metabolic and transcript analysis of the flavonoid pathway in diseased and recovered Nebbiolo and Barbera grapevines (Vitis vinifera L.) following infection by Flavescence dorée phytoplasma. Plant, Cell Environ. 2014; 37: 2183–2200. 10.1111/pce.12332 24689527

[16] Gao R., Wang J., Zhao W., Li X.D., Zhu S.F. and Hao Y.J. Identification of a phytoplasma associated with cherry virescence in China. J. Plant Pathol. 2011; 93: 465–469.

[17] Tan Y., Wei H.R., Wang J.W., Zong X.J., Zhu D.Z. and Liu Q.Z. Phytoplasmas change the source–sink relationship of field-grown sweet cherry by disturbing leaf function. Physiol. Mol. Plant Pathol. 2015; 92: 22–27.

[18] Wang J., Liu Q., Wei W., Davis R.E., Tan Y., Lee M., et al Multilocus genotyping identifies a highly homogeneous phytoplasmal lineage associated with sweet cherry virescence disease in China and its carriage by an erythroneurine leafhopper. Crop Prot. 2018; 106: 13–22.

[19] Wang J., Zhu D., Liu Q., Davis R. E. and Zhao Y. First report of sweet cherry virescence disease in China and its association with infection by a ‘Candidatus Phytoplasma ziziphi’-Related Strain. Plant Dis. 2014; 98: 419 10.1094/PDIS-07-13-0787-PDN 30708425

[20] Tan Y., Wang J., Davis R.E., Wei H., Zong X., Wei W., et al Transcriptome analysis reveals a complex array of differentially expressed genes accompanying a source-to-sink change in phytoplasma-infected sweet cherry leaves. Ann. Appl. Biol. 2019; 175: 69–82.

[21] Chen W., Gong L., Guo Z., Wang W., Zhang H., Liu X., et al A novel integrated method for large-scale detection, identification, and quantification of widely targeted metabolites: application in the study of rice metabolomics. Mol. Plant. 2013; 6: 1769–1780. 10.1093/mp/sst080 23702596

[22] Zhang S., Ying H., Pingcuo G., Wang S., Zhao F., Cui Y., et al Identification of potential metabolites mediating bird’s selective feeding on Prunus mira flowers. BioMed research international 2019; 2019: 1395480 10.1155/2019/1395480 31341887 PMC6612375

[23] Cao H., Ji Y., Li S., Lu L., Tian M., Yang W., et al Extensive metabolic profiles of leaves and stems from the medicinal plant Dendrobium officinale Kimura et Migo. Metabolites 2019; 9: 215. 10.3390/metabo9100215 PMC6835975 31590300

[24] Yang M., Yang J., Su L., Sun K., Li D., Liu Y., et al Metabolic profile analysis and identification of key metabolites during rice seed germination under low-temperature stress. Plant Sci. 2019; 289: 110282 10.1016/j.plantsci.2019.110282 31623771

[25] Thévenot E.A., Roux A., Xu Y., Ezan E. and Junot C. Analysis of the human adult urinary metabolome variations with age, body mass index, and gender by implementing a comprehensive workflow for univariate and OPLS statistical analyses. J. Proteome Res. 2015; 14: 3322–3335. 10.1021/acs.jproteome.5b00354 26088811

[26] Wang S., Tu H., Wan J., Chen W., Liu X., Luo J., et al Spatio-temporal distribution and natural variation of metabolites in citrus fruits. Food Chem. 2016; 199: 8–17. 10.1016/j.foodchem.2015.11.113 26775938

[27] Cambiaghi A., Ferrario M. and Masseroli M. Analysis of metabolomic data: tools, current strategies and future challenges for omics data integration. Brief Bioinform 2016; 18: 498–510. 10.1093/bib/bbw031 27075479

[28] Albertazzi G., Milc J., Caffagni A., Francia E., Roncaglia E., Ferrari F., et al Gene expression in grapevine cultivars in response to Bois Noir phytoplasma infection. Gene expression in grapevine cultivars in response to Bois Noir phytoplasma infection. Plant Sci. 2009; 176: 792–804.

[29] Fan G., Cao X., Zhao Z., and Deng M. Transcriptome analysis of the genes related to the morphological changes of Paulownia tomentosa plantlets infected with phytoplasma. Acta Physiol Plant 2015a; 37: 202.

[30] Fan G., Xu E., Deng M., Zhao Z., and Niu S. Phenylpropanoid metabolism, hormone biosynthesis and signal transduction-related genes play crucial roles in the resistance of Paulownia fortunei to paulownia witches’ broom phytoplasma infection. Genes Genom. 2015b; 37: 913–929.

[31] Fan X.P., Liu W., Qiao Y.S., Shang Y.J., Wang G.P., Tian X., et al Comparative transcriptome analysis of Ziziphus jujuba infected by jujube witches’ broom phytoplasmas. Sci Hortic-Amsterdam 2017; 226: 50–58.

[32] Hren M., Nikolić P., Rotter A., Blejec A., Terrier N., Ravnikar M., et al ’Bois noir’ phytoplasma induces significant reprogramming of the leaf transcriptome in the field grown grapevine. BMC Genomics 2009; 10: 460 10.1186/1471-2164-10-460 19799775 PMC2761425

[33] Mardi M., Karimi Farsad L., Gharechahi J. and Salekdeh G. H. In-depth transcriptome sequencing of Mexican lime trees infected with Candidatus Phytoplasma aurantifolia. PLoS One 2015; 10: e0130425 10.1371/journal.pone.0130425 26132073 PMC4489016

[34] Wang H., Ye X., Li J., Tan B., Chen P., Cheng J., et al Transcriptome profiling analysis revealed co-regulation of multiple pathways in jujube during infection by ‘Candidatus Phytoplasma ziziphi’. Gene 2018; 665: 82–95. 10.1016/j.gene.2018.04.070 29709641

[35] Nejat N., Cahill D.M., Vadamalai G., Ziemann M., Rookes J. and Naderali N. Transcriptomics-based analysis using RNA-Seq of the coconut (Cocos nucifera) leaf in response to yellow decline phytoplasma infection. Mol. Genet. Genomics 2015; 290: 1899–1910. 10.1007/s00438-015-1046-2 25893418

[36] Cao X., Fan G., Dong Y., Zhao Z., Deng M., Wang Z., et al Proteome profiling of paulownia seedlings infected with phytoplasma. Front Plant Sci 2017; 8: 342 10.3389/fpls.2017.00342 28344590 PMC5344924

[37] Ji X., Gai Y., Zheng C. and Mu Z. Comparative proteomic analysis provides new insights into mulberry dwarf responses in mulberry (Morus alba L.). Proteomics 2009; 9: 5328–5339. 10.1002/pmic.200900012 19834890

[38] Margaria P., Abbà S., and Palmano S. Novel aspects of grapevine response to phytoplasma infection investigated by a proteomic and phospho-proteomic approach with data integration into functional networks. BMC Genomics 2013; 14: 38 10.1186/1471-2164-14-38 23327683 PMC3564869

[39] Margaria P. and Palmano S. Response of the Vitis vinifera L. cv. ‘Nebbiolo’ proteome to Flavescence dorée phytoplasma infection. Proteomics 2011; 11: 212–224. 10.1002/pmic.201000409 21204249

[40] Wei Z., Wang Z., Li X., Zhao Z., Deng M., Dong Y., et al Comparative proteomic analysis of Paulownia fortunei response to phytoplasma infection with dimethyl sulfate treatment. Int J Genomics 2017; 2017: 6542075 10.1155/2017/6542075 29038787 PMC5605944

[41] Wang Z., Liu W., Fan G., Zhai X., Zhao Z., Dong Y., et al Quantitative proteome-level analysis of paulownia witches’ broom disease with methyl methane sulfonate assistance reveals diverse metabolic changes during the infection and recovery processes. Peer J 2017; 5: 3495–3495. 10.7717/peerj.3495 28690927 PMC5497676

[42] Taheri F., Nematzadeh G., Zamharir M.G., Nekouei M.K., Naghavi M., Mardi M., et al Proteomic analysis of the Mexican lime tree response to “Candidatus Phytoplasma aurantifolia” infection. Mol. Biosyst. 2011; 7: 3028–3035. 10.1039/c1mb05268c 21853195

[43] Prezelj N., Covington E., Roitsch T., Gruden K., Fragner L., Weckwerth W., et al, 2016. Metabolic consequences of infection of grapevine (Vitis vinifera L.) cv. "Modra frankinja" with flavescence dorée phytoplasma. Front. Plant Sci. 2016; 7: 711 10.3389/fpls.2016.00711 27242887 PMC4876132

[44] Xue C., Liu Z., Dai L., Bu J., Liu M., Zhao Z., et al, 2018. Changing host photosynthetic, carbohydrate, and energy metabolisms play important roles in phytoplasma infection. Phytopathology 2018; 108: 1067–1077. 10.1094/PHYTO-02-18-0058-R 29648946

[45] Gai Y.P., Han X.J., Li Y.Q., Yuan C.Z., Mo Y.Y., Guo F.Y., et al Metabolomic analysis reveals the potential metabolites and pathogenesis involved in mulberry yellow dwarf disease. Plant Cell Environ. 2014; 37: 1474–1490. 10.1111/pce.12255 24329897

[46] Luge T., Kube M., Freiwald A., Meierhofer D., Seemüller E. and Sauer S. Transcriptomics assisted proteomic analysis of Nicotiana occidentalis infected by Candidatus Phytoplasma mali strain AT. Proteomics 2014; 14: 1882–1889. 10.1002/pmic.201300551 24920314

[47] Okita T. W. and Preiss J. Starch degradation in spinach leaves. Plant Physiol. 1980, 66: 870–876. 10.1104/pp.66.5.870 16661544 PMC440744

[48] Delmotte N., Knief C., Chaffron S., Innerebner G., Roschitzki B., Schlapbach R., et al Community proteogenomics reveals insights into the physiology of phyllosphere bacteria. Proc. Natl. Acad. Sci. U.S.A. 2009; 106: 16428–16433. 10.1073/pnas.0905240106 19805315 PMC2738620

[49] Silva Z., Sampaio M.M., Henne A., Böhm A., Gutzat R., Boos W., et al The high-affinity maltose/trehalose ABC transporter in the extremely thermophilic bacterium Thermus thermophilus HB27 also recognizes sucrose and palatinose. J. Bacteriol. 2005; 187, 1210–1218. 10.1128/JB.187.4.1210-1218.2005 15687184 PMC545625

[50] Kube M., Mitrovic J., Duduk B., Rabus R. and Seemüller E. Current view on phytoplasma genomes and encoded metabolism. Sci. World J. 2012; 2012: 185942 10.1100/2012/185942 22550465 PMC3322544

[51] Ding F., Wang M., Zhang S. and Ai X. Changes in SBPase activity influence photosynthetic capacity, growth, and tolerance to chilling stress in transgenic tomato plants. Sci. Rep. 2016; 6: 32741 10.1038/srep32741 27586456 PMC5009361

[52] Wang J., Song L., Jiao Q., Yang S., Gao R., Lu X., et al Comparative genome analysis of jujube witches’-broom Phytoplasma, an obligate pathogen that causes jujube witches’-broom disease. BMC Genomics 2018b; 19: 689–689. 10.1186/s12864-018-5075-1 30231900 PMC6148798

[53] Fischer M. K. and Shingleton A. W. Host plant and ants influence the honeydew sugar composition of aphids. Funct. Ecol. 2001; 15: 544–550.

[54] Rhodes J. D., Croghan P. C. and Dixon A. F. G. Dietary sucrose and oligosaccharide synthesis in relation to osmoregulation in the pea aphid, Acyrthoslphon pisum. Physiol. Entomol. 1997; 22: 373–379.

[55] Mauck K.E., De Moraes C.M. and Mescher M.C. Deceptive chemical signals induced by a plant virus attract insect vectors to inferior hosts. Proceedings of the National Academy of Sciences, 2010; 107(8): 3600–3605. 10.1073/pnas.0907191107 20133719 PMC2840436

[56] Sugio A., MacLean A.M., Grieve V.M. and Hogenhout S.A. Phytoplasma protein effector SAP11 enhances insect vector reproduction by manipulating plant development and defense hormone biosynthesis. Proceedings of the National Academy of Sciences, 2010; 108(48): 1254–1263. 10.1073/pnas.1105664108 PMC3228479 22065743

[57] Michaud M. and Jouhet J. Lipid trafficking at membrane contact sites during plant development and stress response. Front Plant Sci 2019; 10: 2 10.3389/fpls.2019.00002 30713540 PMC6346683

[58] Nishiguchi S., Murata K., Ube N., Ueno K., Tebayashi S.I., Teraishi M., et al Accumulation of 9- and 13-KODEs in response to jasmonic acid treatment and pathogenic infection in rice. J. Pestic. Sci. 2018; 43: 191–197. 10.1584/jpestics.D18-022 30363135 PMC6140683

[59] Feng S., Saw C. L., Lee Y. K. and Huang D. Fungal-stressed germination of black soybeans leads to generation of oxooctadecadienoic acids in addition to glyceollins. J. Agric. Food Chem. 2018; 55: 8589–8595. 10.1021/jf0716735 17892258

[60] Prieto J. A., Ebri A. and Collar C. Composition and distribution of individual molecular species of major glycolipids in wheat flour. J Am Oil Chem So 1992; 69: 1019–1022.

[61] Yuan D., Wu Z. and Wang Y., 2016 Evolution of the diacylglycerol lipases. Prog. Lipid Res. 64, 85–97. 10.1016/j.plipres.2016.08.004 27568643

[62] Munder P. G., Modolell M., Andreesen R., Weltzien H. U. and Westphal O. Lysophosphatidylcholine (lysolecithin) and its synthetic analogues. Immunemodulating and other biologic effects. Springer Semin. Immunopathol. 1979; 2: 187–203.

[63] Kong X., Wei B., Gao Z., Zhou Y., Shi F., Zhou X., et al Changes in membrane lipid composition and function accompanying chilling injury in bell peppers. Plant Cell Physiol. 2018; 59: 167–178. 10.1093/pcp/pcx171 29136239

[64] Condrea E., 1980. Solubilization of human red cell membranes by lysolecithins of various chain lengths. Experientia 1980; 36: 531–533. 10.1007/BF01965781 7379942

[65] Gedvilaite A., Jomantiene R., Dabrisius J., Norkiene M. and Davis R. E. Functional analysis of a lipolytic protein encoded in phytoplasma phage based genomic island. Microbiol. Res. 2014; 169: 388–394. 10.1016/j.micres.2013.08.007 24168924

[66] Sohlenkamp C. and Geiger O. Bacterial membrane lipids: diversity in structures and pathways. FEMS Microbiol. Rev. 2016; 40: 133–159. 10.1093/femsre/fuv008 25862689

[67] Harwood J. L. 1—Plant acyl lipids: structure, distribution, and analysis in Lipids: Structure and Function (ed Stumpf P. K.) 1–55 (Academic Press, 1980).

[68] Christie, W. W. The LipidWeb, https://www.lipidhome.co.uk/index.html.

[69] Hildebrandt T.M., Nesi A.N., Araújo W.L. and Braun H.P. Amino acid catabolism in plants. Mol Plant 2015; 8: 1563–1579. 10.1016/j.molp.2015.09.005 26384576

[70] Swain Tony. Phenolics in the environment. Biochemistry of Plant Phenolics. Springer, Boston, MA, 1979; 617–640.

[71] Camm E. L. and Towers G. H. N. Phenylalanine ammonia lyase. Phytochemistry 1973; 12: 961–973.

[72] Vogt T. Phenylpropanoid biosynthesis. Mol Plant 2010; 3: 2–20. 10.1093/mp/ssp106 20035037

[73] Zhao Y. Auxin biosynthesis and its role in plant development. Annu. Rev. Plant Biol. 2010; 61: 49–64. 10.1146/annurev-arplant-042809-112308 20192736 PMC3070418

[74] Michael A.J. Biosynthesis of polyamines and polyamine-containing molecules. Biochem. J. 2016; 473: 2315 10.1042/BCJ20160185 27470594

[75] Facchini P. J. Alkaloid biosynthesis in plants: biochemistry, cell biology, molecular regulation, and metabolic engineering applications. Annu. Rev. Plant Physiol. Plant Biol. 2001; 52: 29–66. 10.1146/annurev.arplant.52.1.29 11337391

[76] Zeidler D., Zähringer U., Gerber I., Dubery I., Hartung T., Bors W., et al Innate immunity in Arabidopsis thaliana: lipopolysaccharides activate nitric oxide synthase (NOS) and induce defense genes. Proc. Natl. Acad. Sci. U. S. A. 2004; 101: 15811–15816. 10.1073/pnas.0404536101 15498873 PMC524824

[77] Ávila A. C., Ochoa J., Proaño K. and Martínez M. C. Jasmonic acid and nitric oxide protects naranjilla (Solanum quitoense) against infection by Fusarium oxysporum f. sp. quitoense by eliciting plant defense responses. Physiol. Mol. Plant Pathol. 2019; 106: 129–136.

[78] Mur L.A., Santosa I.E., Laarhoven L.J., Holton N.J., Harren F.J. and Smith A.R. Laser photoacoustic detection allows in planta detection of nitric oxide in tobacco following challenge with avirulent and virulent Pseudomonas syringae Pathovars. Plant Physiol. 2005; 138: 1247–12;58. 10.1104/pp.104.055772 16009999 PMC1176398

[79] Prats E., Mur L. A. J., Sanderson R. and Carver T. L. W. Nitric oxide contributes both to papilla-based resistance and the hypersensitive response in barley attacked by Blumeria graminis f. sp. hordei. Mol. Plant Pathol. 2005; 6: 65–78. 10.1111/j.1364-3703.2004.00266.x 20565639

[80] Barroso J.B., Corpas F.J., Carreras A., Sandalio L.M., Valderrama R., Palma J., et al Localization of nitric-oxide synthase in plant peroxisomes. J. Biol. Chem. 1999; 274: 36729–36733. 10.1074/jbc.274.51.36729 10593979

[81] Corpas F. J., Palma J. M., Del Río L. A. and Barroso J. B. Evidence supporting the existence of L-arginine-dependent nitric oxide synthase activity in plants. New Phytol. 2009; 184: 9–14. 10.1111/j.1469-8137.2009.02989.x 19659743

[82] Knowles R. G. and Moncada S., 1994. Nitric oxide synthases in mammals. Biochem. J 1994; 298: 249–258. 10.1042/bj2980249 7510950 PMC1137932

[83] Tsikas D. and Wu G. Homoarginine, arginine, and relatives: analysis, metabolism, transport, physiology, and pathology. Amino Acids 2015; 47: 1697–1702. 10.1007/s00726-015-2055-5 26210755

[84] Bizarro C. V. and Schuck D. C. Purine and pyrimidine nucleotide metabolism in Mollicutes. Genet. Mol. Biol. 2015; 30: 190–201.

[85] Irving H. R. and Gehring C. Molecular methods for the study of signal transduction in plants in Cyclic Nucleotide Signaling in Plants: Methods and Protocols (ed Gehring Chris) 1–11 (Humana Press, 2013). 10.1007/978-1-62703-441-8_1 23681568

[86] Isner J. C. and Maathuis F. J. M. cGMP signalling in plants: from enigma to main stream. Funct. Plant Biol. 2018; 45: 93–101. 10.1071/FP16337 32291024

[87] Meier S., Madeo L., Ederli L., Donaldson L., Pasqualini S. and Gehring C. Deciphering cGMP signatures and cGMP-dependent pathways in plant defence. Plant Signal. Behav. 2009; 4: 307–309. 10.4161/psb.4.4.8066 19794847 PMC2664491

[88] Penson S.P., Schuurink R.C., Fath A., Gubler F., Jacobsen J.V. and Jones R.L. cGMP is required for gibberellic acid-induced gene expression in barley aleurone. The Plant cell 2009; 8: 2325–2333. 10.1105/tpc.8.12.2325 PMC161355 12239379

[89] Isner J. C., Nühse T. and Maathuis F. J. M., 2012. The cyclic nucleotide cGMP is involved in plant hormone signalling and alters phosphorylation of Arabidopsis thaliana root proteins. J. Exp. Bot. 2012; 63: 3199–3205. 10.1093/jxb/ers045 22345640 PMC3350932

[90] Zhao Y., Qi Z. and Berkowitz G. A. Teaching an old hormone new tricks: cytosolic Ca^2+^ elevation involvement in plant brassinosteroid signal transduction cascades. Plant Physiol. 2012; 163: 555–565. 10.1104/pp.112.213371 PMC3793037 23852441

[91] Dermastia M. Plant hormones in phytoplasma infected plants. Front Plant Sci 2019; 10: 477 10.3389/fpls.2019.00477 31057582 PMC6478762

[92] Mierziak J., Kostyn K. and Kulma A. Flavonoids as important molecules of plant interactions with the environment. Molecules 2014; 19: 16240–16265. 10.3390/molecules191016240 25310150 PMC6270724

[93] Stafford H. A. Flavonoid evolution: an enzymic approach. Plant Physiol. 1991; 96: 680–685. 10.1104/pp.96.3.680 16668242 PMC1080830

