# Supplementary material for: Integration of metabolomics and existing omics data reveals new insights into phytoplasma-induced metabolic reprogramming in host plants
Source: PLoS One. 2021 Feb 4;16(2):e0246203. doi: 10.1371/journal.pone.0246203 (PMC7861385; doi:10.1371/journal.pone.0246203)
Supplement: S1 Fig — Color key scale is shown on the right of the heatmap. (PPTX) [file pone.0246203.s001.pptx]

## Slide 1
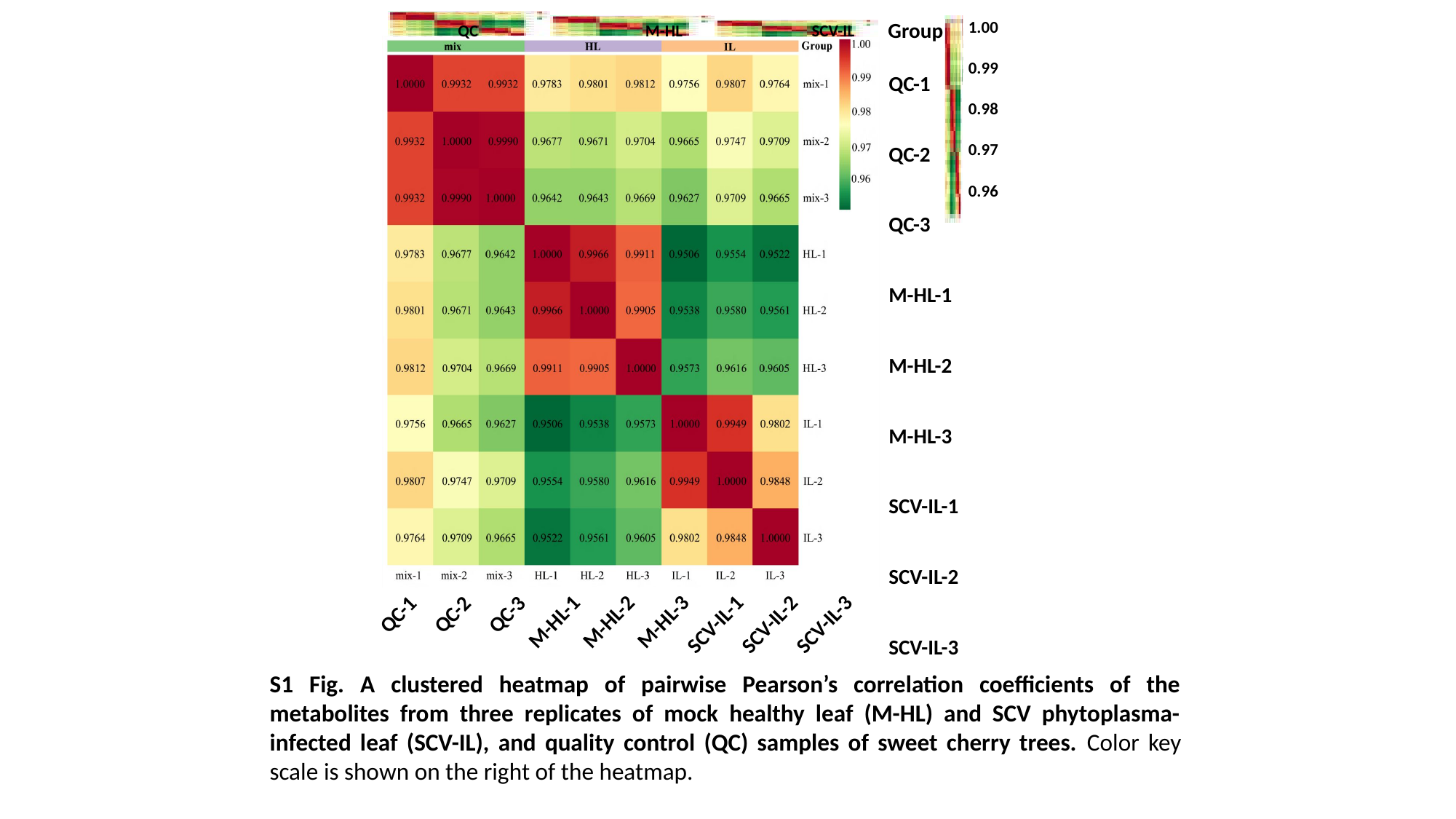

1.00
0.99
0.98
0.97
0.96
Group
QC M-HL SCV-IL
QC-1
QC-2
QC-3
M-HL-1
M-HL-2
M-HL-3
SCV-IL-1
SCV-IL-2
SCV-IL-3
M-HL-1
M-HL-2
M-HL-3
SCV-IL-1
SCV-IL-2
SCV-IL-3
QC-1
QC-2
QC-3
S1 Fig. A clustered heatmap of pairwise Pearson’s correlation coefficients of the metabolites from three replicates of mock healthy leaf (M-HL) and SCV phytoplasma-infected leaf (SCV-IL), and quality control (QC) samples of sweet cherry trees. Color key scale is shown on the right of the heatmap.
